# Interobserver Reliability of the Rotterdam Diabetic Foot Study Test Battery for Assessing Cutaneous Sensibility

**DOI:** 10.1002/dmrr.70251

**Published:** 2026-11-10

**Authors:** Nadine Boers, Isabelle M. L. P. Kamm, Manuel Castro Cabezas, T. M. Fakkel, Willem D. Rinkel, J. Henk Coert

**Affiliations:** ^1^ Department of Plastic, Reconstructive, and Hand Surgery Utrecht University Medical Center Utrecht the Netherlands; ^2^ Department of Internal Medicine/Center for Diabetes, Endocrinology, and Vascular Medicine Franciscus Gasthuis & Vlietland Hospital Rotterdam the Netherlands; ^3^ Department of Internal Medicine and Endocrinology Erasmus University Medical Center Rotterdam the Netherlands; ^4^ Department of Plastic, Reconstructive, and Hand Surgery ZGT/MST Twente the Netherlands

**Keywords:** diabetes mellitus, diabetic polyneuropathy, diagnosis, diagnostic tests, physical examination, sensory thresholds

## Abstract

**Aim:**

The Rotterdam Diabetic Foot (RDF) Test Battery was developed to assess sensory function and predict the risk of diabetic foot ulceration and falls. We evaluated its interobserver reliability in patients with diabetic sensorimotor polyneuropathy.

**Methods:**

The RDF Test Batteries include a continuous and dichotomous 39‐item scale and a shorter dichotomous 13‐item scale. The 39‐item test battery was administered by two investigators, and the 13‐item test battery by five investigators with varying experience. Percentage agreement, Cohen's kappa coefficient (*κ*) and intraclass correlation coefficient (ICC) were calculated, including agreement in ulcer risk classification based on predefined cut‐off values derived from the 39‐item and 13‐item test battery sum scores; incident ulcer outcomes were not collected.

**Results:**

The continuous 39‐item test battery (*n* = 36 test batteries) showed low to high exact agreement (33.9%–100%), poor item‐level ICCs (0.04–0.19), and small median between‐observer differences. Dichotomous 39‐item (*n* = 56) and 13‐item test batteries (*n* = 310) showed low percentage agreement (42.9% and 47.1%) but good reliability (ICC 0.83, 95% CI: 0.67–0.92 and 0.80, 95% CI: 0.73–0.85). Cut‐off based classification of ulcer risk of the 39‐item and 13‐item test batteries showed acceptable to high agreement (96.4% and 87.1%) and substantial reliability (*κ* = 0.67 and 0.73, both *p* < 0.001).

**Conclusion:**

Both instruments showed good reproducibility in sensory staging, although this varied using scoring approach and analysis level. The dichotomous test batteries showed good reliability at the sum‐score level, whereas exact agreement for the continuous 39‐item test battery and reliability of several individual items were limited. Clinical use and ulcer risk classification require further evaluation before routine implementation.

## 1 Introduction

Diabetic sensorimotor polyneuropathy (DSP) is known to lead to major complications, including falls, diabetic foot ulceration, amputation and mortality [1, 2, 3, 4]. Consequently, early diagnosis of DSP is essential and international guidelines emphasise the necessity of screening for sensory loss [5, 6, 7]. Commonly used clinical tests include threshold and innervation density measures [8, 9], but uncertainty remains regarding the optimal test modalities and application sites on feet [5, 6, 7].

The Rotterdam Diabetic Foot (RDF) Test Battery was developed to assess sensory function of the feet using monofilament, two‐point discrimination, vibrations sense, temperature perception and proprioception testing at nerve‐specific test sites [10, 11, 12]. Psychometric validation showed that the test items form a scale, that is certain tests become aberrant before others, including test locations [10, 11]. Results on test items are combined into a summed score, with higher scores reflecting more severe sensory loss. The full 39‐item version is primarily used for research purposes, whereas the shorter 13‐item version is developed for its clinical applicability for screening on sensory loss [11, 13, 14]. Both versions are able to differentiate the diabetic patients at high or low risk for diabetic foot ulceration, based on the degree of sensory loss, with reported sensitivities of 67.5% and 77.5% and specificity of 84.8% and 72.9%, respectively [13].

To date, the interobserver reliability of the RDF Test Batteries has not yet been established, especially when applied by observers with varying levels of clinical experience [15]. This is clinically relevant, as patients are often repeatedly assessed over time by different healthcare professionals. This study assesses the interobserver reliability of both the 39‐item and 13‐item versions, including agreement in cut‐off‐based diabetic foot ulcer risk classification, and carried out in a large cohort of patients with diabetes other than the original RDF Study population.

## 2 Materials and Methods

### 2.1 Study Design

This study was conducted as part of the ongoing DeCompression (DECO) trial [16]. The protocol was approved by the Medical Ethical Committee (MEC 19–335/G), and informed consent was obtained from all participants.

### 2.2 Subjects

Participants were recruited during their initial or subsequent DECO trial related visits to the outpatient clinics of participating hospitals. All patients with type 1 or 2 diabetes exhibiting at least one neuropathic symptom, as assessed by the Michigan Neuropathy Screening Instrument (MNSI), were eligible for inclusion.

### 2.3 The Rotterdam Diabetic Foot Study Test Battery

The Battery tests static and moving two‐point discrimination (static 2PD and moving 2PD), static one‐point discrimination (static 1PD), vibration sense, perception of cold, Romberg's balance test, and comprises information on complaints of numbness and history of foot ulceration or amputation [10, 11, 12]. An overview of all test items and corresponding test sites for both the 39‐item and 13‐item RDF Test Batteries is presented in Figure 1. The 39‐item test battery constitutes of both an original ordinal scoring and dichotomised version. The 13‐item test battery is a shorter, psychometrically validated version with dichotomised (aberrant and non‐aberrant) items, based on reference values [10, 11, 12].

**FIGURE 1:**
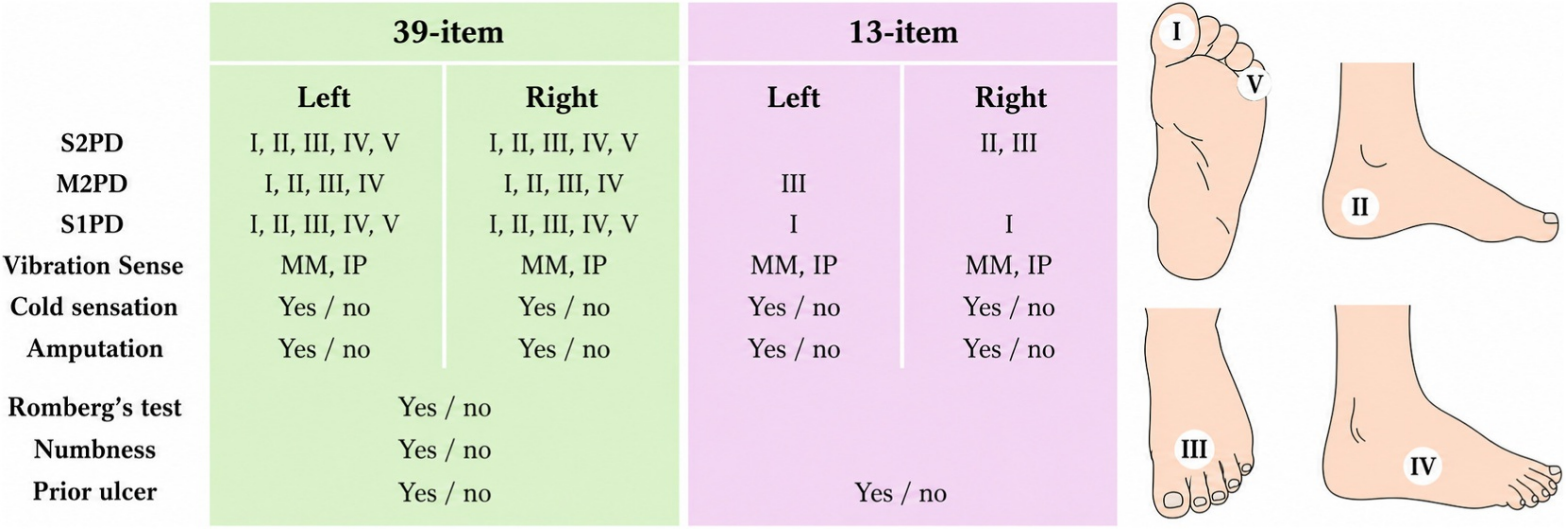
39‐item and 13‐item RDF Study test battery. 2PD, two‐point discrimination; IP, interphalangeal joint; MM, medial malleolus. Roman capitals are indicative of test locations: I, hallux; II, medial heel; III, first dorsal web; IV, lateral foot; and V, fifth toe.

Static 2PD, moving 2PD and static 1PD testing was carried out on five sites in concordance with the nerve territories of the foot. Static and moving 2PD was performed using a Disk‐Criminator (US Neurologicals LLC, Poulsbo, Washington), with distances ranging from 2 to 15 mm. When dichotomised, a cutoff value of 8 mm was used and values greater than 8 mm were considered abnormal [17]. Static 1PD was performed using a 20‐piece Semmes‐Weinstein monofilament set (Baseline Tactile, Minneapolis, Minnesota), ranging from 0.008 to 300 g. When the scores were dichotomised in aberrant and non‐aberrant, a value of 10 g was used, in accordance with current international standards for diabetic medical care [7]. Vibration sense was evaluated using a 64 Hz Rydel‐Seiffer tuning fork (US Neurologicals LLC, Poulsbo, Washington, USA) applied to the dorsum of the interphalangeal joint of the hallux and the medial malleolus. Vibration sense was scored on a continuous scale (0–8, or ‘not perceived’) in all patients across all RDF Test Battery versions and subsequently dichotomised based on age‐dependent normative thresholds [18]. Cold sensation was tested by applying a cold metal object to the medial arch of the foot. Data on perceived ‘numbness’ was retrieved from the MNSI and a history of foot complications from the patient interview. Each test item was scored as normal (0) or abnormal (1) and summed to reflect the overall degree of sensory loss in both feet (39‐item and 13‐item test battery sum scores).

The total score can also be dichotomised using predefined cut‐off values proposed by Rinkel et al., scores of ≥ 24 for the 39‐item and ≥ 7 for the 13‐item test battery indicate high risk for diabetic foot ulcer development, while lower scores indicate low risk (i.e., less sensory loss) [13]. This classification is a categorisation of RDF outcomes based on the extent of sensory loss rather than an observed ulcer outcome. The present study evaluates only whether observers assign the same cut‐off‐based category; no ulcer follow‐up was collected, and the predictive validity of the classification was not assessed.

### 2.4 Data Collection

Observers were selected to represent different levels of experience. The 39‐item RDF Test Battery was executed by two observers: one research‐physician with one year of clinical experience and one research‐physician with no prior experience. The 13‐item RDF Test Battery was executed by five observers working in six different pairs, with experience levels varying from one year to no prior experience. All observers received detailed instructions and a thorough training, including protocol demonstrations, and were blinded to each other's test results. The test battery (continuous 39‐item, dichotomous 39‐item or dichotomous 13‐item) was used was determined by patient availability. The second observer performed a re‐test on the same day following a short interval, with the test order randomized. Additionally, a subset of patients underwent a second 13‐item RDF assessment after an interval of 3–9 months, performed by a different pair of observers. Demographic and clinical data were retrieved from electronic patient records. Assessment duration was not recorded for individual study assessments. In our experience with standardized administration, the continuous 39‐item test battery generally requires approximately 15–30 minutes, depending on the patient's sensory function and the number of stimulus levels required to establish thresholds; more preserved sensibility may require additional measurements. The dichotomous 39‐item and 13‐item test batteries can generally be completed within approximately 10 and 5 minutes, respectively.

### 2.5 Statistical Analyses

Reproducibility was measured by both agreement and reliability, as suggested by De Vet and colleagues [19]. Interobserver agreement reflects measurement precision and was reported as percentage agreement, interpreted as acceptable (0.75–0.90) or high (> 0.90) [20]. Because the scale increments are small, we calculated both absolute agreement and agreement with a margin of 1, 2 and 3 scale points.

Reliability reflects the consistency between observers while correcting for chance agreement. For continuous variables, reliability was assessed using the intraclass correlation coefficient (ICC), and for dichotomous variables, Cohen's kappa (*κ*) was used. ICC values were interpreted as poor (< 0.5), moderate (0.5–0.75), good (0.75–0.90), or excellent (> 0.90) [21]. Cohen's *κ* values were interpreted as poor (< 0), slight (0–0.20), fair (0.21–0.40), moderate (0.41–0.60), substantial (0.61–0.80), and almost perfect (> 0.81) [22, 23].

Given the clustered nature of the continuous data from the 39‐item Test Battery (ten sites per patient, five per foot), measurements were not strictly independent of each other. To address this, ICCs were estimated using multilevel modelling with both fixed‐ and random‐effects linear regression, applying restricted maximum likelihood. The observer was modelled as a fixed effect, while the two clustering levels were included as random effects: (1) left and right foot within the same patient, and (2) test locations within each foot [21, 24]. A second analysis was performed treating the observer as a random effect. Using multilevel modelling, the observer effect (i.e., the difference between the two observers) was estimated, and the ICC was calculated as the proportion of between‐participant variance (*σ*) relative to overall variance, according to the formula: ICC = *σ* ^2^_intercept_/(σ^2^_intercept_ + *σ* ^2^_residual_).

For each paired assessment, the difference between observers was calculated and presented as the median between‐observer difference. The standard error of measurement of continuous variables was used to calculate the smallest detectable difference using the formula: Smallest detectable difference = 1.96·√2·standard error of measurement. The smallest detectable difference represents the smallest change in a measurement that can be interpreted as a true (statistically detectable) change beyond measurement error. Both standard error of measurement and smallest detectable difference values are reported in the original measurement units of the RDF Test Batteries.

To examine potential systematic variation in measurement differences, Bland–Altman plots were used to plot measurement differences against their averages alongside agreement boundaries [25]. Log transformation was applied to non‐parametric data, as recommended by Bland and Altman.

Data are given using percentages and median with interquartile range (IQR). All statistical analyses were performed using IBM SPSS Statistics version 30.0.0, with *p*‐values < 0.05 considered statistically significant.

## 3 Results

A total of 138 patients with symptoms of DSP and varying degrees of sensory loss were included in this study. Patient characteristics are summarised in Table 1, stratified per test battery.

**TABLE 1:** Socio‐demographic and clinical characteristics.

	Continuous 39‐item test battery	Dichotomised 39‐item test battery	Dichotomised 13‐item test battery
Patients, *n*	18	28	119
Test batteries, *n*	36	54	310
Sex, *n* (%)			
Male	11 (61.1)	21 (75)	75 (63)
Female	7 (38.9)	7 (25)	44 (37)
Age in years, median (IQR)	66 (56–76)	68 (57–79)	65 (55–75)
Ethnicity, *n (%)*			
Caucasian	18 (100%)	27 (96.4%)	117 (98.3)
Indo‐Surinamese	0 (0)	0 (0)	1 (0.8)
African	0 (0)	1 (3.6)	0 (0)
Other	0 (0)	0 (0)	1 (0.8)
BMI in kg/m^2^, median (IQR)	27.0 (18.3–35.7)	28.3 (19.8–36.8)	27.1 (20.6–33.6)
Type of diabetes, *n (%)*			
Type 1	5 (27.8)	5 (17.9%)	29 (24.4)
Type 2	13 (72.2)	23 (82.1%)	90 (75.6)
Duration of diabetes in years, median (IQR)	15 (3–27)	18 (4–32)	16 (0–32)
HbA1c in mmol/mol, median (IQR)	58 (43–73)	59 (43–75)	57 (39–75)

Abbreviations: BMI, body mass index; HbA1c, glycated haemoglobin; *n*, number.

**TABLE 2:** Interobserver reliability of the continuous 39‐item RDF test battery in 18 patients.

Test item of the RDF test battery	No of test batteries	No of test items	Median score (IQR)	Absolute agreement (%)	≤ 1 point difference (%)	≤ 2 points difference (%)	≤ 3 points difference (%)
Static 2PD	36	360	> 15 (> 15–> 15)	78.3	86.1	91.7	95.0
Moving 2PD	36	288	> 15 (> 15–> 15)	75.0	84.0	88.2	93.1
Static 1PD	36	360	0.40 (0.16–0.60)	33.9	68.9	85.0	93.3
Vibration sense	36	144	4.5 (2.0–6.0)	59.7	94.4	100	100
Cold perception	36	36		100	100	100	100
Romberg's test	36	36		100	100	100	100

*Note:* All tests included in this table are performed by the same observer pair. The number of test items differ for each test battery: static 2PD = 5 left and 5 right; 10 in each patient; moving; 2PD = 4 left, 4 right; 8 in each patient; static 1PD = 5 left and 5 right; 10 in each patient; and vibration sense = 2 left, 2 right; 4 in each patient.

Abbreviations: 1PD, one‐point discrimination; 2PD, two‐point discrimination; ICC, intraclass correlation coefficient.

### 3.1 Continuous 39‐Item RDF Test Battery

The continuous 39‐item RDF Test Battery was administered by two observers in a subgroup of 18 patients. Bland–Altman plots for the four continuous measurements (static 2PD, moving 2PD, static 1PD and vibration sense) are shown in Supporting Information S1: Figure S1. Table 2 presents the median score and agreement per individual test item. Exact agreement between observers for the separate test items varied from low to high (33.9%–100%). This increased substantially when a margin of 1 point (68.9%–100%), 2 points (85%–100%), and 3 points (93.1%–100%) was chosen, indicating generally good consistency between observers, with small discrepancies, but limited exact agreement for some modalities.

The estimated observer effects were small and not statistically significant (< 0.0 mm for static 2PD, 1.0 mm for moving 2PD, 0.2 g for static 1PD, and 0.1 points for vibration sense). ICCs from the model including ‘observer’ as a fixed effect were 0.10 for static 1PD, 0.09 for static 2PD, 0.19 for moving 2PD and 0.04 for vibration sense, and were comparable to those obtained when ‘observer’ was modelled as a random effect. These ICC values indicate poor agreement at the level of individual test items.

Median paired differences between observers (IQR) were 0 mm (0–0.75) for static 2PD, 0 mm (0–0.75) for moving 2PD, 0.20 g (0.0–0.4) for static 1PD, and 0.0 points (0.0–1.0) for vibration sense. Median test scores for each location are provided in the Supporting Information S1: Tables S1 and S2. The smallest detectible difference was 2.3 mm for static 2PD, 0.4 mm for moving 2PD, 0.9 g for static 1PD and 0.6 points for vibration sense.

### 3.2 Dichotomous RDF Test Batteries

The dichotomous 39‐item and 13‐item test batteries were carried out by two and five observers, respectively, in 28 and 119 patients. This resulted in 56 and 310 pairwise comparisons, respectively.

Table 3 presents the agreement and reliability per individual test item, showing acceptable to high agreement for both the 39‐item (87.5%–100%) and 13‐item scale (84.5%–97.1%). Individual test items showed fair to substantial reliability (Cohen's *κ*: 0.28–0.78 and 0.32–0.81, respectively).

**TABLE 3:** Interobserver reliability of the test items of the dichotomous 39‐ and 13‐item RDF test batteries in 28 and 155 patients.

RDF test battery	No of test batteries	Test item	No of test items	Absolute agreement (%)	Cohen's *κ*	*p*‐value
39‐Item	56	Static 2PD (≤ 8 mm)	540	95.0	0.28	< 0.001
		Moving 2PD (≤ 8 mm)	432	87.5	0.35	< 0.001
		Static 1PD (≤ 10g)	540	95.4	0.60	< 0.001
		Vibration sense	216	89.3	0.78	< 0.001
		Cold perception	54	100		
		Romberg's test	54	100		
13‐Item	310	Static 2PD (≤ 8 mm)	620	91.0	0.32	< 0.001
		Moving 2PD (≤ 8 mm)	310	89.0	0.35	< 0.001
		Static 1PD (≤ 10g)	620	97.1	0.81	< 0.001
		Vibration sense	1240	84.5	0.69	< 0.001

*Note:* The number of test items differ for each test battery. For the 39‐item test battery: static 2PD = 5 left and 5 right; 10 in each patient; moving 2PD = 4 left, 4 right; 8 in each patient; static 1PD = 5 left and 5 right; 10 in each patient; and vibration sense = 2 left, 2 right; 4 in each patient. For the 13‐item test battery: static 2PD = 3 in each patient at non‐related locations; moving 2PD = 1 in each patient; static 1PD = 2 in each patient at non‐related locations; and vibration sense = 2 left, 2 right; 4 in each patient.

Abbreviations: 1PD, one‐point discrimination; 2PD, two‐point discrimination; ICC, intraclass correlation coefficient.

Complete observer agreement on every test item within the test battery was observed in 28.6% of the 39‐item tests and 38.7% of the 13‐item tests. When comparing the sum scores, agreement was higher but still low (42.9% and 47.1%, respectively), with a good reliability (ICC 0.83, 95% CI 0.67–0.92 for the 39‐item; 0.80, 95% CI: 0.73–0.85 for the 13‐item), as presented in Table 4.

**TABLE 4:** Interobserver reliability of the sum score of the dichotomous 39‐ and 13‐item RDF test batteries.

RDF test battery	Observer pair	No of test batteries	Total score Median (IQR)	Absolute agreement on all separate scores (%)	Absolute agreement in sum score (%)	ICC (95% CI) of the sum score
39‐Item	1	56	18.0 (16.0–21.0)	28.6	42.9	0.83 (0.67–0.92)
13‐Item	1	112	7.0 (5.0–9.0)	41.1	44.6	0.86 (0.78–0.92)
	2	76	7.0 (6.0–9.0)	36.8	44.7	0.72 (0.53–0.84)
	3	38	7.5 (5.0–9.0)	47.7	57.9	0.61 (0.22–0.83)
	4	50	7.0 (5.75–9.0)	40.0	52.0	0.87 (0.73–0.94)
	5	34	8.0 (5.75–9.0)	23.5	41.2	0.79 (0.52–0.92)
	Total	310	7.0 (5.0–9.0)	38.7	47.1	0.80 (0.73–0.85)

*Note:* Observer pairs: 1 (NB vs. IK); 2 (NB vs. CM); 3 (NB vs. GB); 4 (NB vs. ED); 5 (IK vs. CM). Levels of experience: one year (NB, ED, GB), three months (CM), and no prior experience (IK).

Abbreviations: ICC, intraclass correlation coefficient; IQR, interquartile range.

The median sum scores for the 39‐item and 13‐item test batteries were 18.0 (16.0–21.0) and 7.0 (5.0–9.0). Median sum scores were comparable across observers (Supporting Information S1: Table S2). The standard error of measurement and smallest detectable difference were 0.48 and 1.32 for the 39‐item sum score, and 0.12 and 0.32 for the 13‐item sum score, respectively.

### 3.3 Pre‐Defined Cut‐Off Values for Future Foot Ulcer Risk

Risk classification based on previously defined cut‐off values showed acceptable to high agreement (96.4% and 87.1%) and substantial reliability (*κ* = 0.65, *p* < 0.001 for the 39‐item; *κ* = 0.73, *p* < 0.001 for the 13‐item) [13]. Across observer pairs, agreement for the 13‐item test battery ranged from acceptable to high (78.9%–92.0%), with Cohen's *κ* indicating moderate to almost perfect reliability (range: 0.55–0.83). Cross‐tabulations are shown in Supporting Information S1: Table S5, showing one discordant classification among 28 paired assessments for the 39‐item test battery and 20 discordant classifications among 155 paired assessments for the 13‐item test battery. These analyses assessed interobserver agreement in predefined risk classification, not predictive validity because no prospective ulcer outcomes were collected.

## 4 Discussion

This study provides a comprehensive psychometric evaluation of the interobserver reliability of three versions of the RDF Test Battery and its individual test items in patients with lower extremity neuropathy. Building on previous evidence supporting the RDF Test Battery for predicting diabetic foot ulceration [13], our findings demonstrate overall good interobserver reliability for sum scores of the dichotomous test batteries, including among observers with different levels of clinical experience, whereas exact agreement for the continuous 39‐item test battery varied widely and individual ICCs were poor. These findings support producible use for some outcomes under standardized, trained conditions, while routine clinical implementation should be further evaluated. When using the continuous 39‐item RDF Test Battery, the broad range of absolute agreement (33.9%–100%) across different subdomains of sensory modalities assessed highlights the difficulty in achieving consistent outcomes across different observers. Several factors may underlie this variability and will be discussed in further detail.

Agreement improved substantially with an allowance for a margin of 1, 2 and 3 points on the scale, indicating clinically relevant general consistency between observers despite minor scoring discrepancies. These differences can be considered minor relative to the number of possible response categories in the full 39‐item RDF scale. For example, static 2PD and moving 2PD each have 15 possible responses (2 to more than 15 mm), while static 1PD has 21 test values (0.008 to more than 300 g) on a logarithmic scale. A one‐step difference may therefore represent a relatively small proportion of the full scale; however, whether such differences are acceptable depends on the intended use, particularly when serial changes in individual patients are interpreted.

Overall, interobserver agreement was good, with a small, non‐significant interobserver effect and small median differences, despite low ICC estimates. This discrepancy is likely explained by the fact that the ICC reflects relative interobserver reliability. This decreases when within‐participant (residual) variance is large, relative to between‐participant variance (i.e., due to range restriction). Therefore, in a clinically heterogenous diabetic population, ICCs should not be interpreted in isolation but alongside absolute agreement, Bland‐Altman plots, the standard error of measurement and the smallest detectable difference. Even in this context, the low ICCs indicate that continuous individual‐item scores should be interpreted cautiously. Also, our findings suggest that, after appropriate training, observers with different levels of experience can reliably apply the RDF Test Battery in a consistent manner. This may support the practical use of the RDF Test Battery in clinical settings under standardized settings, where patients are often assessed over time by different healthcare professionals.

At the aggregated item level, Semmes‐Weinstein monofilaments (static 1PD) showed the lowest exact agreement (33.9%). This is likely explained by the logarithmic structure of the monofilament scale and the distribution of scores in our cohort, with most patients scoring at the lower end of the scale (< 2 g in 89%). In this range, small differences between observers may result in different recorded monofilament values, thereby reducing exact agreement despite limited absolute differences. The median between‐observer difference (0.4 g, IQR 0.16–0.60) remained small and below both the estimated smallest detectable difference (0.90 g) and previously reported minimal clinically important differences of 1.1 g by Rinkel et al., and 0.91 points by Collings et al. [17, 26], indicating that between observer differences were generally small. In addition, tingling and pain during the physical examination as well as prolonged sensory testing (up to 30 minutes for the full battery) in patients with DSP may provoke irritation or fatigue and may introduce response inconsistency [27]. These factors suggest that exact agreement is less informative, but the magnitude of disagreement is. However, the low exact agreement still limits interpretation of small changes in individual patients.

In contrast, dichotomous static 1PD outcomes showed substantially higher agreement (95.4% and 97.1% for the 39‐ and 13‐item test batteries, respectively), with reliability ranging from moderate to near‐perfect (*κ* = 0.60 and 0.81). This is consistent with previous studies reporting moderate to high reliability of the 10‐g monofilament in patients with diabetes [15, 28, 29, 30]. Together, these findings suggest that dichotomous scoring is not only easier to apply and more patient‐friendly in clinical practice but also more robust than measurements on a continuous scale. In this cohort, dichotomous scoring was therefore more reproducible than continuous threshold measurement. Its relative simplicity and shorter administration time (5–10 minutes vs. 15–30 minutes) may favour routine clinical use, especially in time‐constrained clinical settings, although feasibility and implementation were not directly evaluated.

For two‐point discrimination (static 2PD and moving 2PD), the continuous measures showed acceptable interobserver agreement (78.3% and 75.0%, respectively). This may be partly explained by the linear millimetre scale, which makes exact agreement less sensitive to small observer‐related differences than the logarithmic monofilament scale, although variation in application pressure remains a potential source of measurement variability. Dichotomised outcomes yielded even higher agreement (87.5%–95.0%), but only fair *κ* values (0.28–0.35). This apparent discrepancy is likely explained by the highly skewed distribution of outcomes in our cohort, with about 90% of participants classified in the same category (> 15 mm). In such situations, percentage agreement may be high, while Cohen's *κ* is reduced because it corrects for chance agreement and is sensitive to imbalanced marginal distributions. Accordingly, the relatively low *κ* values likely reflect outcome imbalance rather than clinically meaningful disagreement between observers.

For vibration sense in the continuous 39‐item test battery, the absolute agreement was modest (59.7%) but increased markedly when a 1‐point margin was allowed (94.4%), with a median between‐observer difference below the beforementioned smallest detectable difference. Dichotomised outcomes showed high and acceptable agreement in both the 39‐item and 13‐item test batteries (89.3% and 84.5%, respectively), with substantial reliability (*κ* = 0.78 and 0.69). These estimates are consistent with earlier reports in patients with DSP (*κ* = 0.54, 95% CI 0.37–0.73) [15]. In our cohort, median vibration scores (4.5, IQR 2.0–6.0) were close to the age‐adjusted dichotomisation thresholds (≥ 3.5–≥ 4.5), meaning that small measurement differences could shift category assignment and reduce observed agreement. The high percentage agreement is encouraging, but the lower exact agreement and threshold sensitivity warrant caution when interpreting serial measurements in individual patients.

Cold perception and Romberg's tests showed perfect interobserver agreement. This contrasts with the poor reliability for cold perception reported by Wasan et al. [31], likely reflecting differences in test design. While their protocol required graded comparison with a control site (better/worse/different/same), the RDF test batteries use a simple binary classification (normal vs. abnormal), which likely improves both feasibility and reproducibility.

At the sum‐score level, exact agreement was low for the dichotomous 39‐item and 13‐item RDF test batteries (42.9% and 47.1%), indicating that observers frequently did not obtain identical total scores. Exact agreement requires both observers to obtain the same sum score; therefore, a difference in only one dichotomous item already counts as a disagreement for the entire assessment. In contrast, the good ICCs (0.83 and 0.80) indicate that between‐patient differences were large relative to between‐observer measurement error and that observers generally differentiated patients with lower and higher levels of sensory loss in a similar manner. This is further supported by the small differences between observer pairs in the 13‐item battery, despite differences in experience. Thus, although the test batteries showed good relative reliability, individual sum scores were not fully interchangeable between observers and small differences should be interpreted cautiously. Importantly, most differences in sum score did not cross the predefined cut‐off and therefore did not alter the assigned risk category. Accordingly, agreement was high to acceptable for cut‐off–based diabetic foot ulcer risk classification (96.4% for the 39‐item and 87.1% for the 13‐item version). For the 39‐item test battery, the lower *κ* relative to the high percentage agreement resulted from the strongly imbalanced risk‐category distribution, which increased the expected chance agreement. Under these circumstances, categorisation was generally reproducible. However, further evaluation in a more heterogenous group of patients is necessary before widespread implementation. Also, these reflect the reproducibility of category assignment rather than predictive validity or clinical utility.

Our study has several limitations. First, the Bland‐Altman analyses for static 2PD and moving 2PD suggested larger between‐observer differences at higher mean scores, with some indication of systematic bias. This underscores the need for careful standardisation of measurement procedures, even if these differences are unlikely to substantially affect the clinical use of the composite sum scores. Second, our study population may not fully reflect the broader population in which the RDF Test Battery is intended to be used. Because 89% of participants had preserved static 1PD (< 2 g), our cohort was particularly suited for evaluating interobserver reliability in the context of screening for early sensory loss; however, future studies should confirm these findings in more heterogeneous populations with more advanced sensory loss. Third, since no follow‐up data on ulcer development were available in this study population, the findings on ulcer risk classification should be seen in light of the scoring system and not as validation of the prediction model itself. Fourth, members of the author group were involved in the development of the RDF Test Battery, creating a potential for interpretive bias. Although the analyses were prespecified to mitigate this risk, independent replication by investigators not involved in its development and further evaluation in routine clinical practice are warranted.

## 5 Conclusion

Although exact item‐level agreement for the continuous 39‐item RDF Test Battery varied between observers, most discrepancies were limited to one scoring step, indicating good interobserver consistency. The dichotomous 39‐item and 13‐item RDF Test Batteries demonstrated good interobserver reliability at the sum‐score level, with substantial reproducibility for cut‐off–based diabetic foot ulcer risk classification. Together, these findings support the reliability of the RDF Test Batteries across observers, including those with different levels of experience. Given its shorter administration time, the 13‐item RDF Test Battery appears more feasible for clinical practice, although implementation studies are warranted.

## Author Contributions

**Nadine Boers:** conceptualisation, data curation, formal analysis, methodology, project administration, visualisation, writing – original draft preparation (leading). **Isabelle M. L. P. Kamm:** data curation, methodology, project administration, visualisation, writing – original draft preparation (support). **Manuel Castro Cabezas:** funding acquisition, writing – review and editing. **T. M. Fakkel:** funding acquisition, methodology, writing – review and editing. **Willem D. Rinkel:** funding acquisition, supervision, writing – review and editing. **J. Henk Coert:** funding acquisition, supervision, writing – review and editing.

## Funding

This work was supported by ZonMw (Netherlands Organisation for Health Research and Development), grant number 852001906. ZonMw's reviewers of the DECO trial project proposal did suggestions on trial design and methods of analysis, but do not have influence on data nor interpretation of this study. Sponsor: Utrecht University Medical Centre.

## Ethics Statement

The protocol was approved by the Medical Ethical Committee (MEC 19–335/G), and informed consent was obtained from all included participants.

## Conflicts of Interest

The authors declare no conflicts of interest.

## Supporting information


Supporting Information S1

## Data Availability

The data that support the findings of this study are available on request from the corresponding author. The data are not publicly available due to privacy or ethical restrictions.
