# Supplementary material for: Interobserver Reliability of the Rotterdam Diabetic Foot Study Test Battery for Assessing Cutaneous Sensibility
Source: Diabetes Metab Res Rev. 2026 Nov 10;42(8):e70251. doi: 10.1002/dmrr.70251 (PMC13750436; doi:10.1002/dmrr.70251)
Supplement: Supplementary file 1 [file DMRR-42-e70251-s001.docx]

**Supplementary File**

**Figure 1.** Bland-Altman plots of the continuous measurements of the RDF-39 test battery

*The middle line shows the mean difference, together with upper and lower limits of agreement. For the S1PD, logarithmic transformation was used. Abbreviations: M2PD, moving two-point discrimination; S1PD, static one-point discrimination; S2PD, static two-point discrimination*

**Table 1.** Median scores, differences and the smallest detectable changes of the continuous 39-item RDF test battery in 18 patients

| Test item of the RDF test battery | N of test batteries | Median score (IQR) | Median rater difference (IQR) | Standard error of the mean | Smallest detectable difference |
| --- | --- | --- | --- | --- | --- |
| Static 2PD | 36 | >15 (>15->15) | 0 (0-0.75) | 0.33 | 0.90 |
| Moving 2PD | 36 | >15 (>15->15) | 0 (0-0.75) | 0.82 | 2.27 |
| Static 1PD (gram) | 36 | 0.40 (0.16-0.60) | 0.20 (0.00-0.40) | 0.13 | 0.37 |
| Vibration sense | 36 | 4.5 (2.0-6.0) | 0.0 (0.0-1.0) | 0.21 | 0.57 |

*All tests included in this table are performed by the same rater pair. Static 2PD and moving 2PD include a range from 0 to 15 and >15. Static 1PD include stepwise outcomes on a logarithmic scale from 0.008 to 100, given in grams. Vibration sense includes a range from 0 to 8 on a continuous scale.*

*Abbreviations: IP, interphalangeal joint; MM, medial malleolus; 2PD, two-point discrimination.*

**Table 2.** Multilevel modelling outcomes of the continuous 39-item RDF test battery in 18 patients

| Test item of the RDF test battery | N of test batteries | Rater effect | P-value of rater effect | ICC  fixed-effect analyses | ICC  random-effect analyses |
| --- | --- | --- | --- | --- | --- |
| Static 2PD | 36 | -0.16 | 0.61 | 0.10 | 0.07 |
| Moving 2PD | 36 | <0.00 | 1.00 | 0.09 | 0.07 |
| Sstatic 1PD (gram) | 36 | 0.97 | 0.69 | 0.19 | 0.07 |
| Vibration sense | 36 | 0.07 | 0.87 | 0.04 | 0.00 |

*All tests included in this table are performed by the same rater pair. Static 2PD and moving 2PD include a range from 0 to 15 and >15. Static 1PD include stepwise outcomes on a logarithmic scale from 0.008 to 100, given in grams. Vibration sense includes a range from 0 to 8 on a continuous scale. The*

*Abbreviations: IP, interphalangeal joint; MM, medial malleolus; 2PD, two-point discrimination.*

**Table 3.** Median scores for separate test items at separate locations of the continuous 39-item RDF test battery in 18 patients

| Test item of the RDF test battery | N of test batteries | Side | Median score (IQR) | | | | | | |
| --- | --- | --- | --- | --- | --- | --- | --- | --- | --- |
|  |  |  | *Location I* | *Location II* | *Location III* | *Location IV* | *Location V* | *At the MM* | *At the IP* |
| Static 2PD | 36 | *Left* | >15 (13->15) | >15 (>15->15) | >15 (>15->15) | >15 (>15->15) | >15 (>15->15) | . | . |
|  |  | *Right* | >15 (15->15) | >15 (>15->15) | >15 (>15->15) | >15 (>15->15) | >15 (>15->15) | . | . |
| Moving 2PD | 36 | *Left* | >15 (10->15) | >15 (>15->15) | >15 (15->15) | >15 (>15->15) | . | . | . |
|  |  | *Right* | >15 (11->15) | >15 (>15->15) | >15 (15->15) | >15 (>15->15) | . | . | . |
| Static 1PD (gram) | 36 | *Left* | 0.60 (0.22-1.85) | 0.40 (0.16-0.60) | 0.16 (0.04-0.60) | 0.40 (0.04-0.90) | 0.60 (0.40-4.00) | . | . |
|  |  | *Right* | 0.40 (0.40-1.00) | 0.40 (0.16-0.40) | 0.16 (0.03-0.60) | 0.40 (0.16-0.60) | 0.60 (0.40-1.40) | . | . |
| Vibration sense | 36 | *Left* | . | . | . | . | . | 5.0 (2.25-6.0) | 3.5 (0.0-5.0) |
|  |  | *Right* | . | . | . | . | . | 6.0 (2.25-6.0) | 4.0 (0.0-5.0) |

*All tests included in this table are performed by the same rater pair. S2PD and M2PD include a range from 0 to 15 and >15. S1PD include stepwise outcomes on a logarithmic scale from 0.008 to 100, given in grams. Vibration sense includes a range from 0 to 8 on a continuous scale.*

*Abbreviations: IP, interphalangeal joint; MM, medial malleolus; 2PD, two-point discrimination. Roman capitals are indicatives of test locations: I, hallux; II, medial heel; III, first dorsal web; IV, lateral foot and V, fifth toe.*

**Table 4**. Median sum scores and differences between raters of the dichotomous 39- and 13-item RDF test batteries

| RDF test battery | Rater pair | N of tests | Sum score  Median (IQR) | Rater difference  Median (IQR) | Standard error of the mean | Smallest detectable difference |
| --- | --- | --- | --- | --- | --- | --- |
| 39-item | 1 | 56 | 18.0 (16.0-21.0) | 1.0 (0.0-2.0) | 0.48 | 1.32 |
| 13-item | 1 | 112 | 7.0 (5.0-9.0) | 1.0 (0.0-1.0) | . | . |
|  | 2 | 76 | 7.0 (6.0-9.0) | 1.0 (0.0-2.0) | . | . |
|  | 3 | 38 | 7.5 (5.0-9.0) | 0.0 (0.0-1.0) | . | . |
|  | 4 | 50 | 7.0 (5.75-9.0) | 0.0 (0.0-1.0) | . | . |
|  | 5 | 34 | 8.0 (5.75-9.0) | 1.0 (0.0-2.0) | . | . |
|  | Total | 155 | 7.0 (5.0-9.0) | . | 0.12 | 0.32 |

*Rater pair 1 (NB vs IK); rater pair 2 (NB vs CM); rater pair 3 (NB vs GB); rater pair 4 (NB vs ED); rater pair 4 (IK vs CM). Abbreviations: IQR, interquartile range; 2PD, two-point discrimination.*

**Table 5**. Cross-tabulations and interobserver reliability of cut-off-based diabetic foot ulcer risk classification for the 39- and 13-item test batteries

| **Observer pair** | **Individual test batteries, n** | **First observer classification** | **Second observer classification, n** | | **Row total, n** | **Absolute agreement, n (%)** | **Cohen's κ** | ***p*-value** |
| --- | --- | --- | --- | --- | --- | --- | --- | --- |
|  |  |  | **Low risk** | **High risk** |  |  |  |  |
| **39-item test battery** | | | | | | | | |
| 1 | 56 | Low risk | 26 | 1 | 27 | 27 (96.4) | 0.65 | <0.001 |
|  |  | High risk | 0 | 1 | 1 |  |  |  |
|  |  | **Total** | **26** | **2** | **28** |  |  |  |
| **13-item test battery** | | | | | | | | |
| 1 | 112 | Low risk | 22 | 2 | 24 | 50 (89.3) | 0.78 | <0.001 |
|  |  | High risk | 4 | 28 | 32 |  |  |  |
|  |  | **Total** | **26** | **30** | **56** |  |  |  |
| 2 | 76 | Low risk | 9 | 2 | 11 | 33 (86.8) | 0.69 | <0.001 |
|  |  | High risk | 3 | 24 | 27 |  |  |  |
|  |  | **Total** | **12** | **26** | **38** |  |  |  |
| 3 | 38 | Low risk | 5 | 2 | 7 | 15 (78.9) | 0.55 | 0.017 |
|  |  | High risk | 2 | 10 | 12 |  |  |  |
|  |  | **Total** | **7** | **12** | **19** |  |  |  |
| 4 | 50 | Low risk | 9 | 0 | 9 | 23 (92.0) | 0.83 | <0.001 |
|  |  | High risk | 2 | 14 | 16 |  |  |  |
|  |  | **Total** | **11** | **14** | **25** |  |  |  |
| 5 | 34 | Low risk | 4 | 3 | 7 | 14 (82.4) | 0.61 | 0.006 |
|  |  | High risk | 0 | 10 | 10 |  |  |  |
|  |  | **Total** | **4** | **13** | **17** |  |  |  |
| **Total** | **310** | **Low risk** | **49** | **9** | **58** | **135 (87.1)** | **0.73** | **<0.001** |
|  |  | **High risk** | **11** | **86** | **97** |  |  |  |
|  |  | **Total** | **60** | **95** | **155** |  |  |  |

*Values are numbers of paired assessments unless otherwise indicated. For the 39-item test battery, sum scores <24 were classified as low risk and scores ≥24 as high risk; for the 13-item test battery, sum scores <7 were classified as low risk and scores ≥7 as high risk. Rows represent classifications assigned by the first observer and columns those assigned by the second observer. Observer pairs: 1, NB versus IK; 2, NB versus CM; 3, NB versus GB; 4, NB versus ED; and 5, IK versus CM. For the 39-item test battery, 56 individual test batteries correspond to 28 paired assessments.*
